# Photophysical and Photocatalytic Properties of BiSnSbO_6_ under Visible Light Irradiation

**DOI:** 10.3390/ma11040491

**Published:** 2018-03-26

**Authors:** Jingfei Luan, Panqi Huang

**Affiliations:** 1 School of Chemistry and Environmental Engineering, Changchun University of Science and Technology, Changchun 130022, China; 2 State Key Laboratory of Pollution Control and Resource Reuse, School of the Environment, Nanjing University, Nanjing 210093, China; 18851070626@126.com

**Keywords:** BiSnSbO_6_, benzotriazole, rhodamine B, photocatalytic degradation, visible light irradiation

## Abstract

BiSnSbO_6_ with strong photocatalytic activity was first fabricated by a high-temperature, solid-state sintering method. The resulting BiSnSbO_6_ was characterized by scanning electron microscopy (SEM), transmission electron microscopy (TEM), X-ray diffraction (XRD), UV-vis diffuse reflectance spectroscopy (DRS) and X-ray photoelectron spectroscopy (XPS). The results showed that BiSnSbO_6_, with a pyrochlore structure and a cubic crystal system by a space group Fd3m, was well crystallized. The lattice parameter or the band gap of BiSnSbO_6_ was 10.234594 Å or 2.83 eV. Compared with N-doped TiO_2_, BiSnSbO_6_ showed higher photocatalytic activity in the degradation of benzotriazole and rhodamine B. The apparent first-order rate constant for BiSnSbO_6_ in the degradation of benzotriazole and rhodamine B was 0.0182 min^−1^ and 0.0147 min^−1^, respectively. On the basis of the scavenger experiment, during the photocatalytic process, the main active species were arranged in order of increasing photodegradation rate: •OH < •O_2_^−^ < h^+^. The removal rate of benzotriazole or rhodamine B was approximately estimated to be 100% with BiSnSbO_6_ as a photocatalyst after 200 min visible-light irradiation. Plentiful CO_2_ produced by the experiment indicated that benzotriazole or rhodamine B was continuously mineralized during the photocatalytic process. Finally, the possible photodegradation pathways of benzotriazole and rhodamine B were deduced.

## 1. Introduction

Benzotriazole UV stabilizers (BZT-UVs) are one of most important synthetic compounds in plastic additives. Because of their large annual production, as well as their widespread use in building materials and paints, environmental and health risk assessments of BZT-UVs have gradually attracted increasing attention. In the current study, most of the target BZT-UVs have been included in the USEPA (United States Environmental Protection Agency) High Production Volume Challenge Program, and the HPV (High Production Volume) list for the Organization for Economic Co-operation and Development, and are each manufactured at over 450 tons and 1000 tons per year [1]. In addition, a substantial part of BZT-UVs have been measured in environmental samples—such as in surface water in rivers and lakes [2,3], soil [4,5], and fish [6,7]—which may result in the different degrees of bioaccumulation of BZT-UVs in an aquatic food web and finally in the human body. It is noteworthy that previous studies have shown the potential for endocrine disruption and liver toxicity by BZT-UVs [3,8]. Nowadays, the removal and mineralization of BZT-UVs have become more difficult, because of its high water solubility, low vapor pressure, low octanol–water distribution coefficients and fair resistance to biodegradation [9,10,11].

TiO_2_, an efficient photocatalyst, has drawn much attention due to its widespread use in self-cleaning construction materials, environmental protection technologies and other applications, but its catalytic activity is limited by ultraviolet (UV) light irradiation [12,13,14,15]. In recent years, there are only two studies advancing the photocatalytic activity of TiO_2_ particles on degrading BZT-UVs. Ding et al. [16] reported that the degradation removal of benzotriazole was significant, up to 89.8% under 180 min UV irradiation when a potential bias of +0.8 V was applied to the TiO_2_ thin film, indicating a synergistic reaction between photocatalysis and electrochemical oxidation. The TiO_2_ loaded on Fe^II^Fe_2_^III^O_4_@C, synthesized by Jorfi et al. [17], effectively acted as an activator of peroxomonosulfate to oxidatively degrade benzotriazole. Another study reported by Xu et al. [11] also studied the impact of other efficient photocatalysts on degrading BZT-UVs. They found that BiOBr catalysts with different morphologies gradually removed nearly 90% of benzotriazole after visible light irradiation for 3 h, whereas P25 only removed about 50% of benzotriazole under the same conditions.

At present, in order to optimize visible light, which occupies 43% of sunlight, many efforts have been made to develop photocatalysis with high activity for visible light. Some new types of semiconductors with an obvious visible light response have been explored in recent years. In addition, elemental doping [18,19] and the formation of heterojunctions [20,21,22,23,24,25,26] have attracted researchers’ attention in order to improve the degradation efficiency of pollutants under visible light irradiation. Among these visible-light-driven photocatalysts, Bi-based photocatalysts with high photocatalytic activity, such as BiVO_4_ [27], BiOX (X = Br, I, Cl) [28,29] and Bi_2_MoO_6_ [30], have been widely used due to their wide range of visible light absorption and environmental friendliness. In addition, it has been found that the visible light absorption of photocatalysts can be enhanced via doping Sb [31]. Yang et al. [32] evaluated the photocatalytic activity of the reactions for CO_2_ reduction and gaseous iso-isopropanol oxidation using a novel Sb-doped SnO_2_/porous g-C_3_N_4_ photocatalyst. The obtained results showed that the prepared samples significantly improved the photocatalytic efficiency. Nasser et al. [33] reported that the energy change caused by Sb doping obviously led to the decrease of the band gap energy of ZnO. Moreover, previous works have shown that the photo-excitation of electrons in an O2p and Bi6s hybrid orbital may result in a charge transfer occurring to a d orbital of the other metal in the composite oxide [34,35,36]. Sn-Sb-Bi composite oxides may respond to visible light irradiation because Sn and Sb respectively occupy 5d orbits in the ground state. Based on above research and discussion, BiSnSbO_6_ was synthesized in this work to measure the photocatalytic degradation of BZT-UVs.

Rhodamine B (RhB), one of the most important elements of xanthene dyes, is resistant to biodegradation and direct photolysis, producing potentially carcinogenic aromatic amines after undergoing natural reductive anaerobic degradation [37,38,39,40]. However, it is widely used in the industrial field, especially as a photosensitizer, quantum counter and active medium in dye lasers [41,42,43]. In this report, therefore, RhB and benzotriazole (BZT) were used as the target contaminants to evaluate the photocatalytic activity of BiSnSbO_6_ with a xenon arc lamp as a visible light source.

The aim of this work was to prepare a new semiconductor catalyst BiSnSbO_6_ via a solid-state reaction method and evaluate the catalytic performance for the photodegradation of RhB and BZT. For comparison, we chose a conventional photocatalyst, nitrogen-doped titanium dioxide (N-doped TiO_2_), as a catalyst for the degradation of RhB and BZT under visible light irradiation. Additionally, the structure and photocatalytic properties of BiSnSbO_6_ have been researched via a variety of techniques, including XRD, SEM, TEM, XPS and UV–vis DRS. Finally, the possible degradation pathways of BZT and RhB have been proposed based on the identification of the reaction intermediates via liquid chromatograph-mass spectrometer (LC–MS) analysis.

## 2. Materials and Methods

### 2.1. Preparation of BiSnSbO_6_ and N-Doped TiO_2_ Photocatalysts

BiSnSbO_6_ was prepared by a high-temperature, solid-state sintering method. Bi_2_O_3_, SnO_2_ and Sb_2_O_5_ with a purity of 99.99% (Sinopharm Group Chemical Reagent Co., Ltd., Shanghai, China) were used as raw materials. Due to the volatile performance of Bi_2_O_3_ at high temperature, we ultimately made a resolve to increase the quantity of Bi_2_O_3_ up to 150%. These fully-mixed materials (*n*(Bi_2_O_3_):*n*(SnO_2_):*n*(Sb_2_O_5_) = 1.5:2:1) were ground in a ball mill until the powder particle size was 1–2 µm. All powders were dried at 200 °C for 4 h before synthesis. Then these powders were mixed in an aluminum oxide crucible (Shenyang Crucible Co., Ltd., Shenyang, China) in which above-mentioned materials were pressed into disks and were sintered in an electric furnace (KSL 1700X, Hefei Kejing Materials Technology CO., Ltd., Hefei, China) at 400 °C for 8 h. After crushing and pressing, the mixture was sintered again in an electric furnace at 900 °C for 25 h. Finally, pure BiSnSbO_6_ catalyst with a light yellow color was obtained after total grinding.

Nitrogen-doped titanium dioxide (N-doped TiO_2_) catalyst was synthesized by a sol-gel method and tetrabutyl titanate was selected as the titanium source. Firstly, 17 mL tetrabutyl titanate and 40 mL absolute ethyl alcohol were mixed as solution A. Then 10 mL glacial acetic acid and 5 mL double distilled water were sequentially dripped into solution A, and then the solution formed a transparent colloidal suspension named solution B after stirring for 0.5 h. Next, aqua ammonia with n(N)/n(Ti) of 8% was added into solution B with vigorous stirring for 1 h. After two days, a xerogel took shape. Eventually, after grinding the xerogel into powder and calcining at 500 °C for 2 h, N-doped TiO_2_ powder could be obtained by grinding these powders in agate mortar and screening by shaker.

### 2.2. Characterization

The particle morphology and chemical composition of BiSnSbO_6_ were obtained respectively by using a transmission electron microscope (TEM, Tecnal F20 S-Twin, FEI Corporation, Hillsboro, OR, USA) and scanning electron microscope-X-ray energy dispersion spectrum (SEM-EDS, LEO 1530VP, LEO Corporation, Dresden, Germany). The oxygen content, Bi^3+^ content, Sn^4+^ content and Sb^5+^ content of BiSnSbO_6_ were analyzed by X-ray photoelectron spectroscopy (XPS, ESCALABMK-2, VG Scientific Ltd., London, UK). In the depth profile of BiSnSbO_6_, its chemical composition was gauged by the argon ion denudation or XPS. The powder X-ray diffraction (XRD) spectra was recorded on a D/MAX-RB advance spectrometer (D/MAX-RB, Rigaku Corporation, Tokyo, Japan) with Cu *K*α radiation (*λ* = 1.54056 Å), in which the operating temperature was 295 K and the scanning range was 2*θ* = 10°–95°. The Brunauer–Emmett–Teller (BET) surface area of BiSnSbO_6_ was determined via a ASAP 2020 physical adsorption apparatus (Micromeritics Corporation, Atlanta, GA, USA) with N_2_ adsorption at liquid nitrogen temperature. The optical properties of BiSnSbO_6_ were estimated by UV-visible spectrophotometer assembled with an integrating sphere (Shimadzu UV-2550 UV-Visible spectrometer, Kyoto, Japan). Room temperature photoluminescence (PL) measurement was performed by using a fluorescence spectrophotometer (Perkin Elmer, LS 55, Waltham, MA, USA).

### 2.3. Photocatalytic Properties Test

A total of 0.8 g photocatalyst (BiSnSbO_6_ or N-doped TiO_2_) powder was added into 300 mL 0.032 mM RhB solution (or 300 mL 0.032 mM BZT solution) in a pyrex glass cell (Jiangsu Yancheng Huaou Industry, Yancheng, China) with a magnetic rotor to form a suspension system. In order to guarantee the establishment of an adsorption/desorption equilibrium among the photocatalyst (BiSnSbO_6_ or N-doped TiO_2_), the RhB dye (or BZT) and dissolved oxygen, above suspension was magnetically stirred in the dark for 45 min before irradiation with simulated sunlight. The photodegradation of RhB (or BZT) (Tianjin Kermel Chemical Reagent Co., Ltd., Tianjin, China) was in progress. The photocatalytic reaction system (Nanjing JYZCPST CO., Ltd., Nanjing, China) that we used in this paper was made up of a 500 W xenon arc lamp of which the major emission wavelength was 436 nm, a cut-off filter with a cut-off wavelength of 400 nm (Jiangsu Nantong JSOL Corporation, Nantong, China), and a magnetic stirrer. The xenon arc lamp which was placed in the internal part of an optical quartz reactor vessel with a diameter of 5.8 cm and a length of 68 cm, was encircled by a quartz jacket, in which some pyrex glass cells with a homogeneous suspension solution of RhB (or BZT) and photocatalyst (BiSnSbO_6_ or N-doped TiO_2_) was circulated. Room temperature (25 °C) was maintained by an outer recycling water glass sleeve. The solution was continuously stirred and inflated. A total of 3 mL sample was sampled at different time intervals and also filtered by using a 0.22 μm filter membrane. The incident photon flux *I_o_*, which was gauged by a radiometer (Model FZ-A, Photoelectric Instrument Factory Beijing Normal University, Beijing, China), was measured to be 4.76 × 10^−6^ Einstein L^−1^·s^−1^ under visible light irradiation (wavelength coverage at 400–700 nm). The incident photon flux on the photoreactor was determined by the distance from the photoreactor to the xenon arc lamp. In addition, the initial pH value in this work was 7.0. The concentration of RhB was measured by an UV-Vis spectrophotometer (Shimadzu UV-2550 UV-Visible spectrometer, Kyoto, Japan) with the detecting wavelength at 553.5 nm. The concentration of BZT was evaluated by Agilent 1200 high performance liquid chromatography (Agilent Technologies, Palo Alto, CA, USA) with an UV detector and a Zorbax 300SB-C18 column (4.6 mm × 150 mm, 5 μm). A mixture of 50% CH_3_CN and 50% distilled deionized water was used as the mobile phase. The UV detection wavelengths were set at 254 nm. The injection volume of post-photodegradation BZT solution was 10 μL and the flow rate was 1 mL·min^−1^.

An ion chromatograph (DX-300, Dionex Corporation, Sunnyvale, CA, USA) was used to analyze the inorganic products generated in the process of RhB and BZT photodegradation. LC-MS (Thermo Quest LCQ Duo, Thermo Fisher Scientific Corporation, Silicon Valley, CA, USA, Beta Basic-C_18_ HPLC column: 150 mm × 2.1 mm, ID of 5 μm, Finnigan, Thermo Fisher Scientific Corporation, Silicon Valley, CA, USA) was applied to measure the appraisal of RhB or BZT and its photodegradation intermediate products. Here, the volume of post-photodegradation solution injected automatically into the LC-MS system was 20 μL. The mixture of 60% methanol and 40% water was served as eluent with a flow rate of 0.2 mL·min^−1^. MS conditions included an electrospray ionization interface, a capillary temperature of 300.15 K with a voltage of 19.00 V, a spray voltage of 5000 V and a constant sheath gas flow rate. Under the negative ion scan mode with the *m*/*z* range of 50–600, the spectrum could be acquired. The intersmat^TM^ IGC120-MB gas chromatograph (Thermo Separation Products Corporation, Brussels, Belgium) assembled with a porapack Q column (length of 3 m and an inner diameter in 0.25 cm), which was linked to a catharometer detector, was used to analyze the CO_2_ production. A total organic carbon (TOC) analyzer (TOC-5000, Shimadzu Corporation, Kyoto, Japan) was used to measure the TOC concentration during the photodegradation of RhB or BZT. The photon efficiency of the photocatalyst (BiSnSbO_6_ or N-doped TiO_2_) was calculated on the basis of the following equation [44,45]:

ϕ = R/*I_o_*

where ϕ was the photon efficiency (%), R was the photodegradation rate of RhB (or BZT) (moL·L^−1^·s^−1^), and *I_o_* was the incident photon flux (Einstein·L^−^^1^·s^−^^1^).

### 2.4. Photoelectrochemical Properties Test

In order to explore the photoelectrochemical properties of BiSnSbO_6_, the modified electrodes were prepared as reported by Mao et al. [46] and the preparation technology was as following: firstly, 2 cm × 1 cm ITO glass was sequentially cleaned by methylbenzene, acetone, ethyl alcohol and deionized water before photoelectrochemical measurement. Secondly, 20 mg of BiSnSbO_6_ powder was mixed in 2 mL of dimethylformamide solution under sonication for 2 h to form the slurry. Thirdly, the slurry was uniformly daubed onto the conductive surface of the ITO substrate and dried at 120 °C for 10 min. The electrochemical measurements were then carried out on a CHI760E electrochemical workstation (Shanghai Chen Hua Instrument Co., Ltd., Shanghai, China) three-electrode photoelectrochemical cell in 0.2 M Na_2_SO_4_ aqueous. Here, the electrolyte with BiSnSbO_6_ coated on ITO glass was as the working electrode, and Ag/AgCl (3 M KCl) was as the counter electrode and a saturated calomel electrode was as the reference electrode. A 500 W xenon arc lamp was utilized as the light source.

## 3. Results and Discussion

### 3.1. Characterization

**Figure 1:**
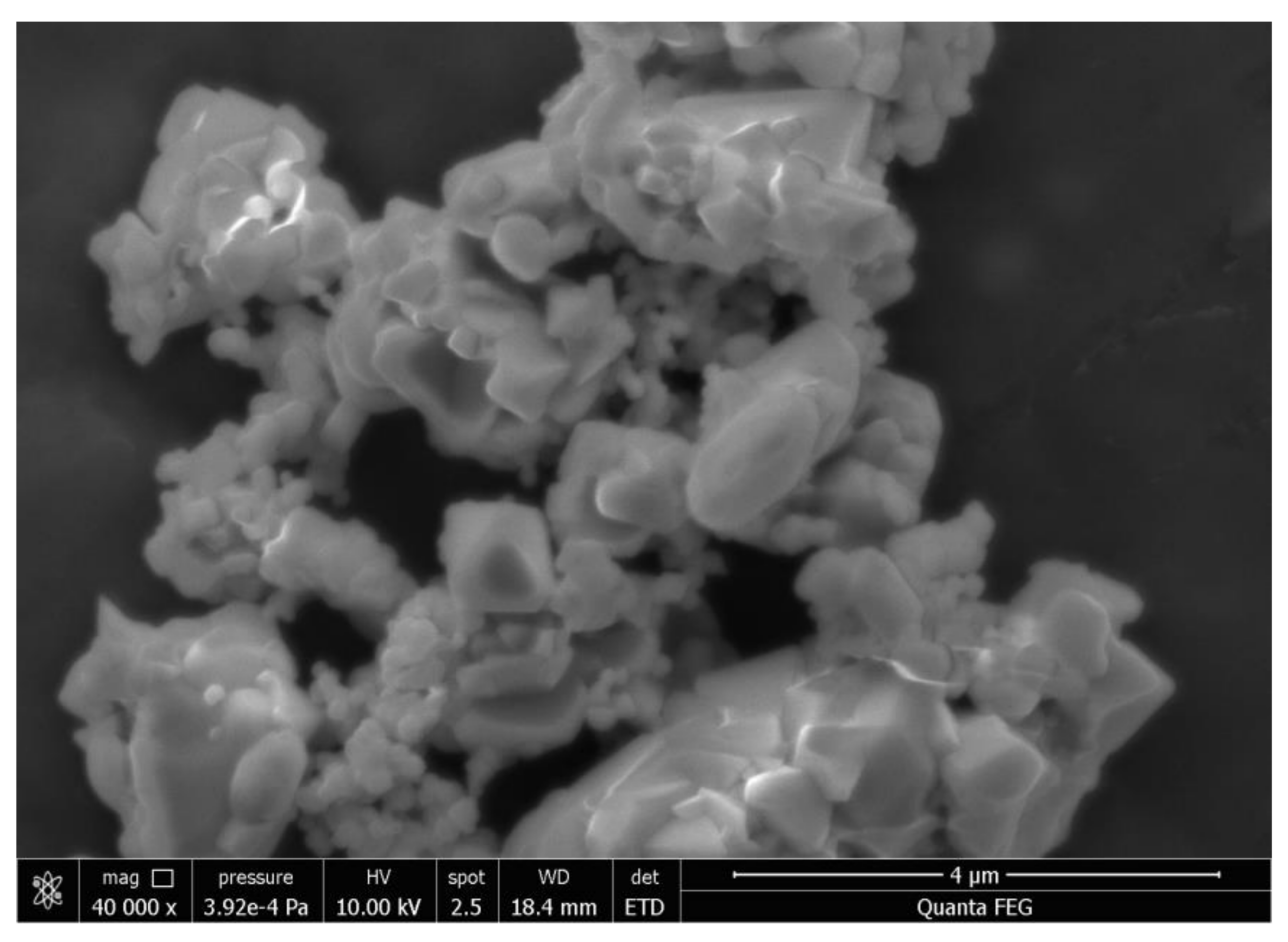
SEM image of BiSnSbO_6_.

**Figure 2:**
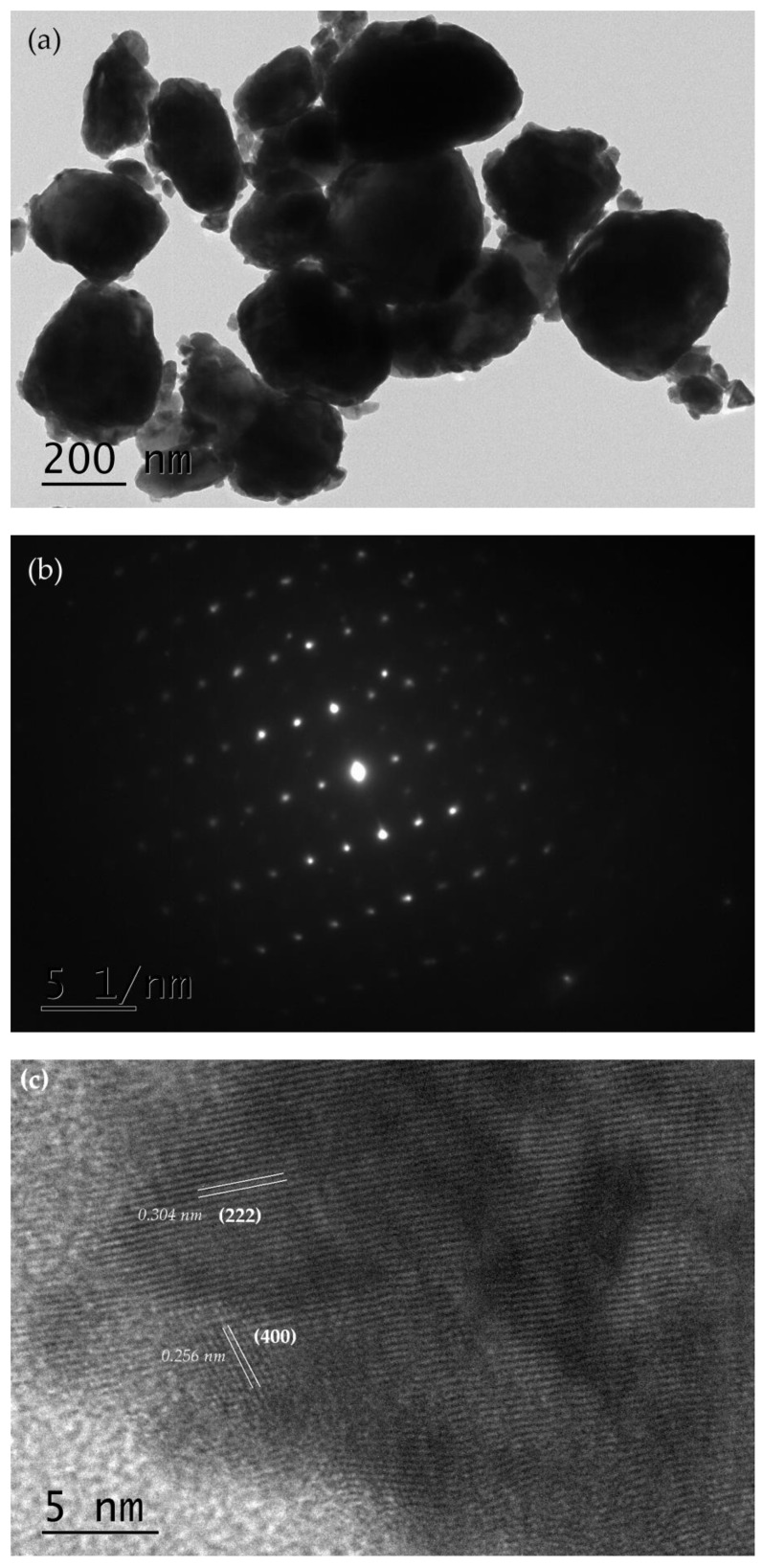
The high resolution TEM picture of BiSnSbO_6_ (**a**), the selected area electron diffraction pattern of BiSnSbO_6_ (**b**) and the HRTEM image of BiSnSbO_6_ with clear lattice fringe spacing (**c**).

In order to obtain insight concerning the microstructure and morphology of BiSnSbO_6_, chemical analysis was carried out by using SEM and TEM. Figure 1 shows the SEM image of BiSnSbO_6_. As shown in Figure 1, the morphology of BiSnSbO_6_ was irregular and the diameter of the particles was about 0.5–1.5 µm. Figure 2a and Figure 2b show a high resolution TEM picture of BiSnSbO_6_ and the selected area electron diffraction pattern of BiSnSbO_6_. It was clear from Figure 2a that the particle size of BiSnSbO_6_ with irregularity was relatively uniform, and the particle size was about 200 nm to 300 nm. Some particles were agglomerated, and this result was similar to the SEM analysis. In Figure 2b, bright diffraction can be observed and the diffraction point is in a cycle arrangement, indicating the highly crystalline nature and single crystal of BiSnSbO_6_. Figure 2c shows the HRTEM image of BiSnSbO_6_ with clear lattice fringe spacing. The lattice fringe spacing shown in Figure 2c was estimated to be 0.304 nm and 0.256 nm, corresponding to the (222) and (400) lattice plane of BiSnSbO_6_, respectively.

**Figure 3:**
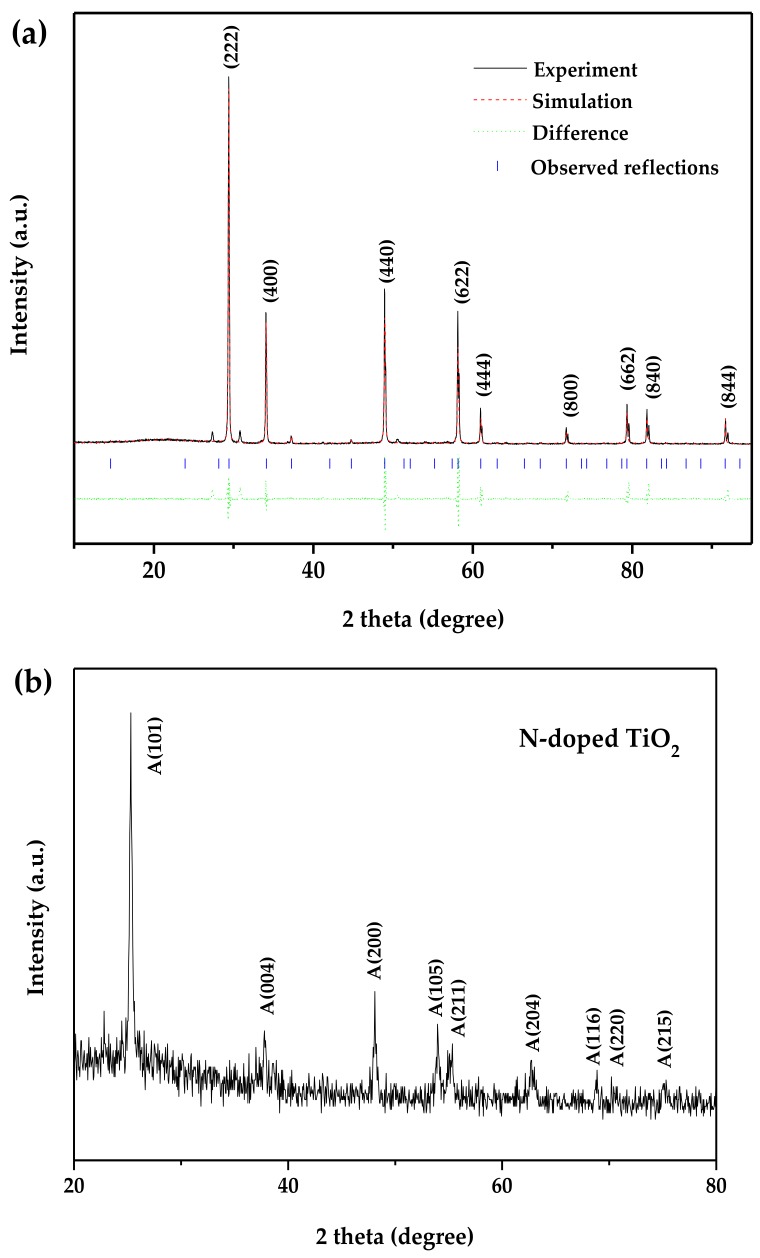
(**a**) The Pawley refinement outcomes of XRD data for BiSnSbO_6_; (**b**) XRD spectrum of N-doped TiO_2_.

According to the Rietveld analysis, the comprehensive structure refinement of the data gathered from the RIETAN^TM^ [47] program and the XRD image of BiSnSbO_6_ are revealed in Figure 3a. The results indicated that BiSnSbO_6_, with a pyrochlore structure and a cubic system with a space group of Fd3m, was well crystallized. Meanwhile, it could be perceived from the XRD image that a good correlation was confirmed between the observed and calculational intensities of the structure of BiSnSbO_6_. The calculation results for BiSnSbO_6_ refinement received the unweighted R factors (*R*_P_ = 15.43% < 20%), revealing good agreement with the experimental pattern in the whole 2θ range of 10°–95°. According to the high-purity precursors adopted in this work, we implied that the slightly refined structure of BiSnSbO_6_ could obtain slightly higher *R* factors. It should be noted that the defects or the disorder/order of a small portion of the atoms could lead to structural varieties, particularly for bond moment distributions, thermal displacement parameters and occupation factors for certain atoms [48]. Moreover, Figure 3b shows the XRD spectrum of N-doped TiO_2_. It could be seen from Figure 3b that the peaks for the as-prepared N-doped TiO_2_ sample corresponded to the anatase TiO_2_ phase according to the JCPDS No. 21-1272.

In addition, BiSnSbO_6_ was a single phase and the lattice parameter α for BiSnSbO_6_ was 10.234594 Å according to above refinement results of the XRD data. The Bragg equation (2dsinθ = nλ, here, n = 1, λ = 1.54056 Å) and the calculation formula for the cubic crystal face spacing equation (d = α(h^2^ + k^2^ + l^2^)^−1/2^) were combined to calculate the lattice parameter α for BiSnSbO_6_. Here, d was the face spacing, and θ was the diffraction angle, and h, k and l were the indices of crystallographic plane. On the basis of the lattice fringe spacing shown in Figure 2c, the lattice parameter α for BiSnSbO_6_ was found to be 10.530869 Å or 10.240000 Å, which was basically accordant to above refined lattice parameter result. According to the calculation of the lattice parameter for every diffraction peak of the XRD data and the refinement results of BiSnSbO_6_, an optimal lattice parameter for BiSnSbO_6_ could be clearly received.

On the basis of the lattice parameter α for BiSnSbO_6_ with a cubic system, the unit cell bulk *V* value of BiSnSbO_6_ was found to be 1072.04(2) Å^3^, which was 2.2 times than that of Bi_2_WO_6_ (*V* = 487.30(5) Å^3^) [49], and 7.8 times than that of N-doped TiO_2_ (*V* =136.57 Å^3^) [50]. Compared with N-doped TiO_2_, the size expansion of BiSnSbO_6_ might result in an increase of the bond lengths. Previous work had found that the closer the M-O-M bond angle was to 180°, the more delocalized the excited state was, which could lead to the easier movement of charge carriers in the matrix [49,50,51]. The increase of the photon efficiency of BiSnSbO_6_ was owing to high diffusivity, which contributed to more excited photogenerated electrons and holes on the surface of a photocatalyst. In addition, the O-Bi-O bond angle of BiSnSbO_6_ was 90°, while the O-Bi-O bond angle of Bi_2_WO_6_ only ranged from 65.5(7)° to 87.3(6)°. As for BiSnSbO_6_, the angle of the Sn-O-Bi bond or Bi-Sn-Sn bond or Bi-O-Bi bond was 116.5(6)° or 135° or 126.8(7)°, which was close to 180°. Moreover, the angle of the Bi-O-Sn bond or Sn-O-Sn bond or O-Sn-O bond was 180°. Therefore, the photocatalytic activity of BiSnSbO_6_ might be accordingly higher.

**Figure 4:**
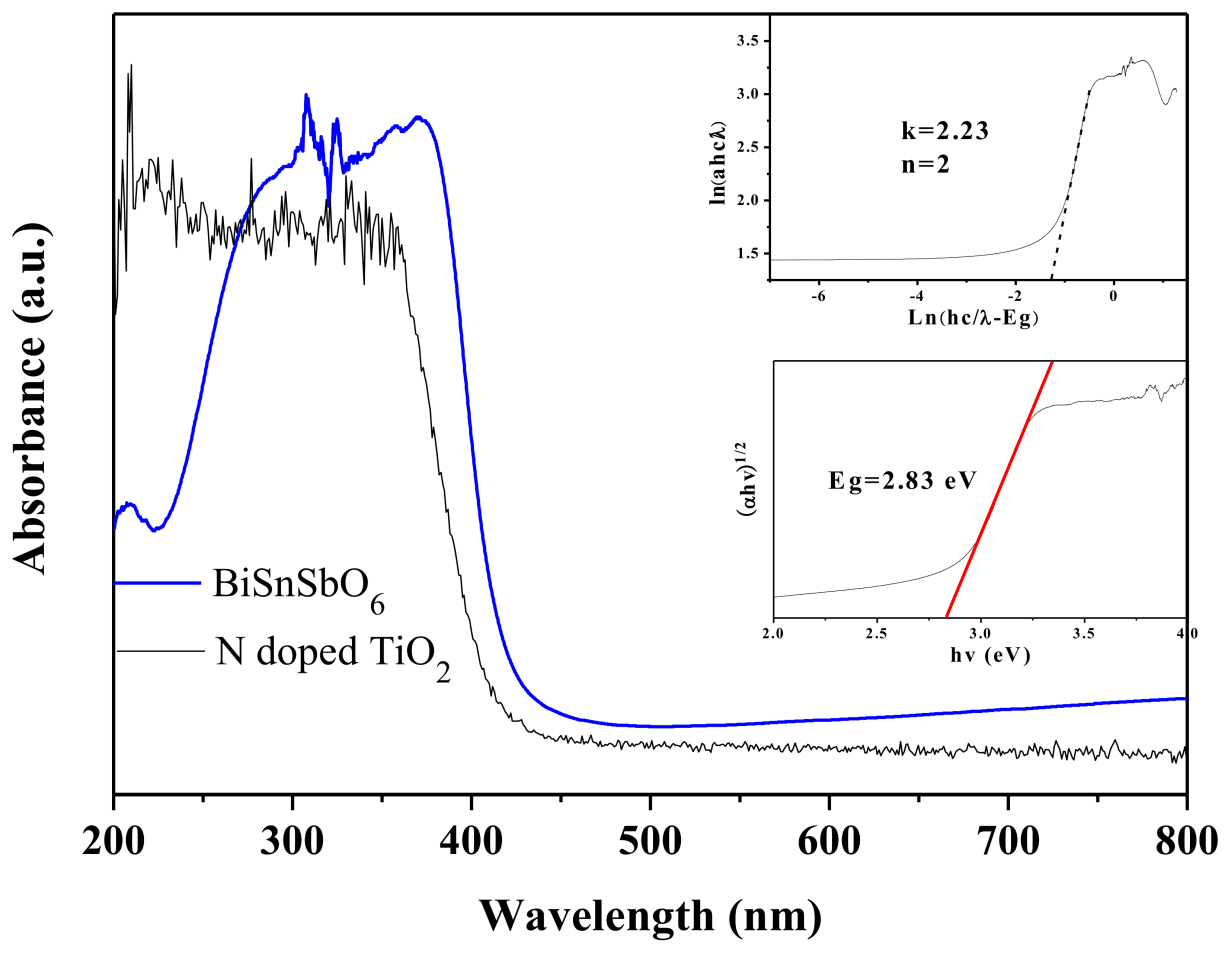
UV-Vis diffuse reflectance spectra of BiSnSbO_6_ and N-doped TiO_2_ (inset was plot of ln(αhν) versus ln(*hν − E_g_*) or plot of (*αhν*)^1/2^ versus *hν* for BiSnSbO_6_).

The absorption spectra of BiSnSbO_6_ and N-doped TiO_2_ are shown in Figure 4. In comparison with the N-doped TiO_2_, of which the photoresponse wavelength was less than 410 nm, the photoresponse wavelength of newly synthesized BiSnSbO_6_ was invented at 440 nm, indicating that BiSnSbO_6_ photocatalyst could response to visible light and rise in the range of the whole absorption spectrum.

The light absorption near the band edge for a crystalline semiconductor was found according to the following equation [52,53]: αhν = A(hν − E_g_)^n^, where A, α, E_g_ and ν were proportional constant, absorption coefficient, band gap and light frequency, respectively. Within this equation, the peculiarity of the interim in a crystalline semiconductor was decided by the superscript n. The following steps were to count E_g_ and n: (i) assuming an approximate value of E_g_ through the equation E_g_ = 1240/λ; (ii) drawing plot ln(αhν) versus ln(hν − E_g_) spectra to obtain the value of n; (iii) drawing plot (αhν)^1/n^ versus hν to refine the value of E_g_. Additionally, hν was equal to ch/λ, where c, h and λ were light velocity, Planck constant and wavelength of incident light. The E_g_ of BiSnSbO_6_ was found to be 2.83 eV in this way. The n of BiSnSbO_6_ was found to be 2, indicating the indirect light transition for BiSnSbO_6_.

**Figure 5:**
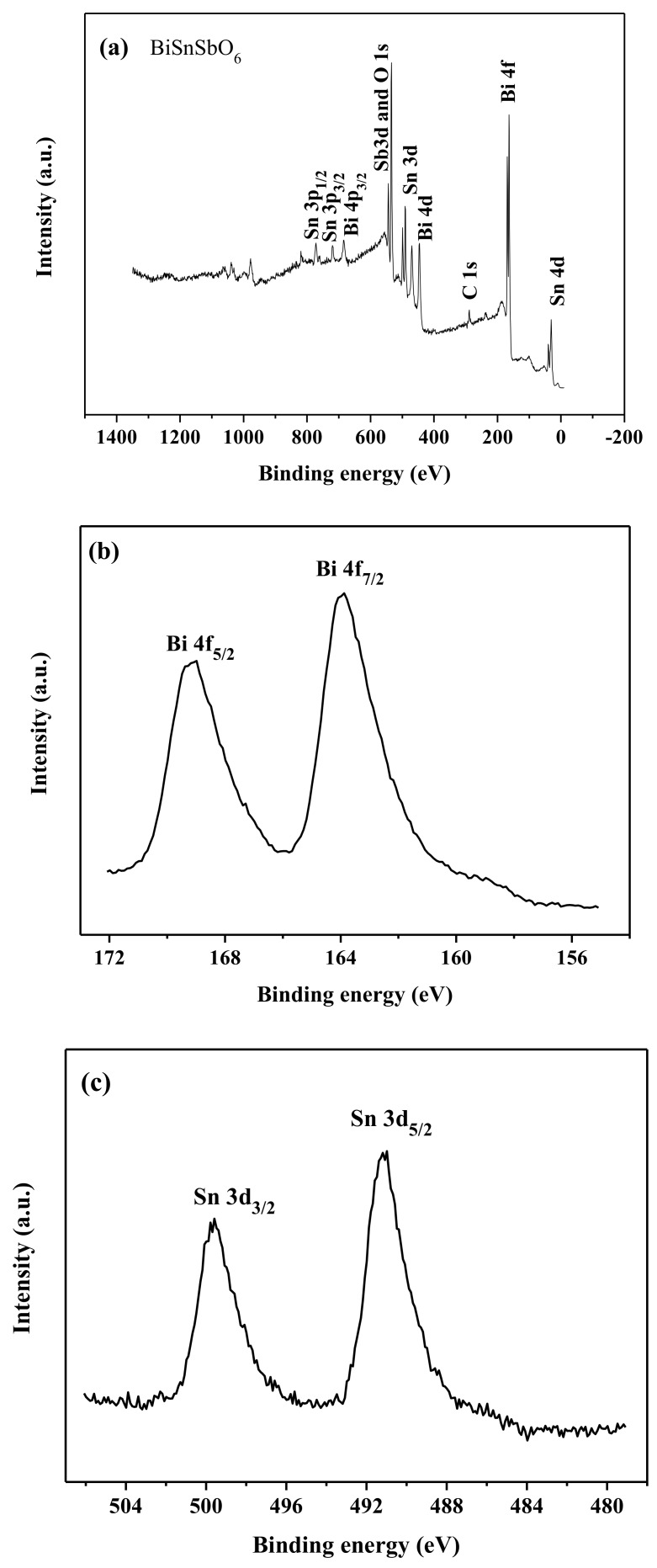

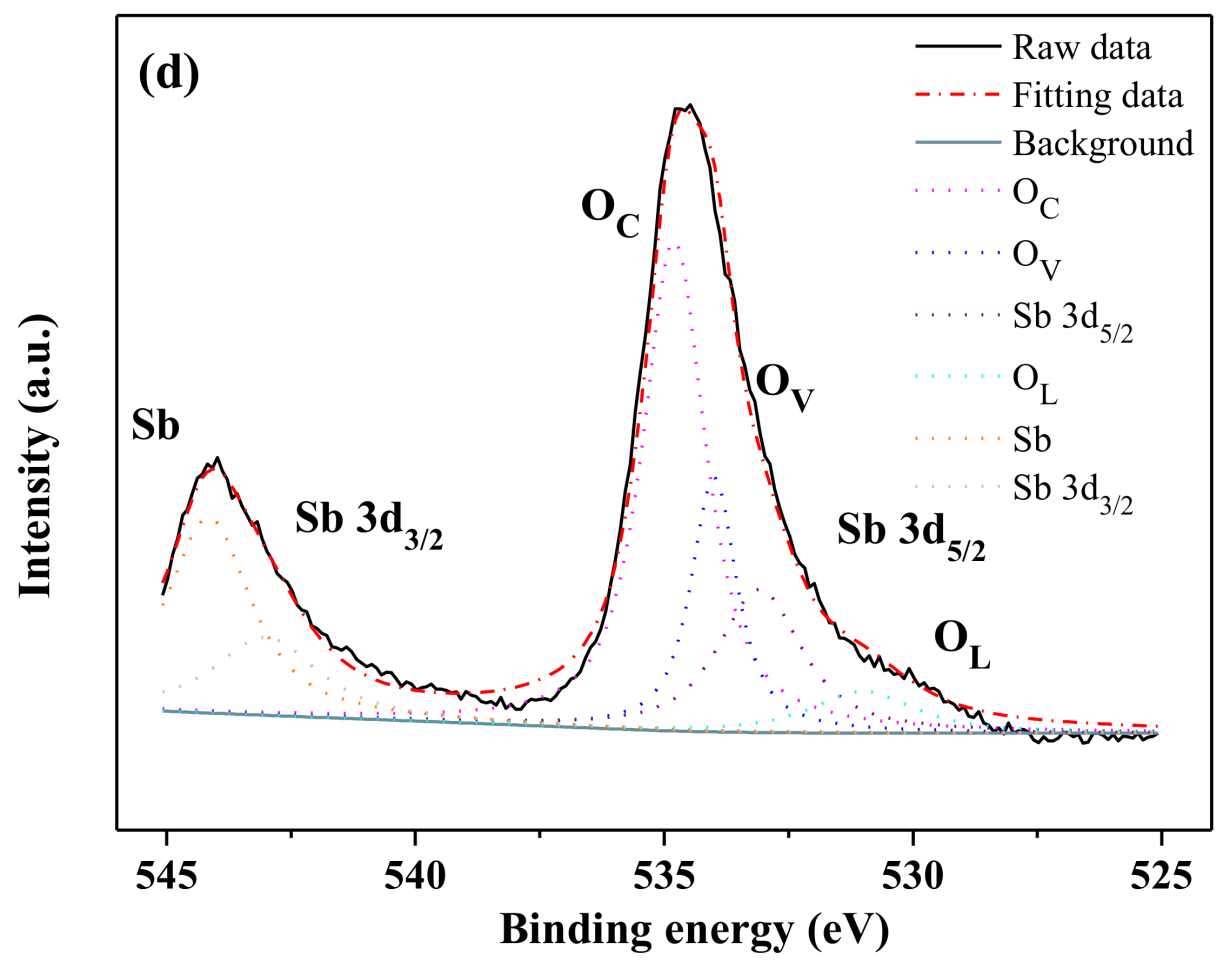
The XPS spectra of BiSnSbO_6_ and every element in BiSnSbO_6_. The full spectrum of BiSnSbO_6_ (**a**); Bi 4f (**b**); Sn 3d (**c**); Sb 3d and O1s (**d**).

The XPS full spectrum of BiSnSbO_6_ and the characteristic peak XPS spectra of their elements are shown in Figure 5. From Figure 5a, it could be seen that the BiSnSbO_6_ catalyst contained only the elements bismuth, stannum, antimony, oxygen and carbon, indicting the absence of any other impurity elements. Here, the carbon element was due to the addition of hydrocarbons which were used to facilitate the testing and calibration of the elements. Hence, it could be easily found that the prepared substance BiSnSbO_6_ was of high purity. Moreover, the XPS peaks of BiSnSbO_6_ showed side-by-side peaks between Sb and O. In order to further analyze the surface chemical states of BiSnSbO_6_, the binding energy of the characteristic peaks of each element in BiSnSbO_6_ are set out in Table 1. The binding energies obtained through the XPS analysis were corrected by referencing the C 1 s line to 285.0 eV. The binding energies of Bi_4f7/2_, Sn_3d5/2_, Sb_3d5/2_ and O_1s_ were 159.80, 486.90, 529.70 and 530.70 eV respectively after correcting C. By comparing the XPS standard binding energy data table and the chemical shifts of each element, the valence state of each element in BiSnSbO_6_ was determined and the oxidation state of Bi, Sn, Sb and O ions from BiSnSbO_6_ were +3, +4, +5 and −2, respectively. In addition, the mean atomic ratio of Bi: Sn: Sb: O measured by the XPS data was 1.00: 0.98: 1.02: 5.98, which was near the theoretical value for BiSnSbO_6_.

### 3.2. Photocatalytic Activity

**Figure 6:**
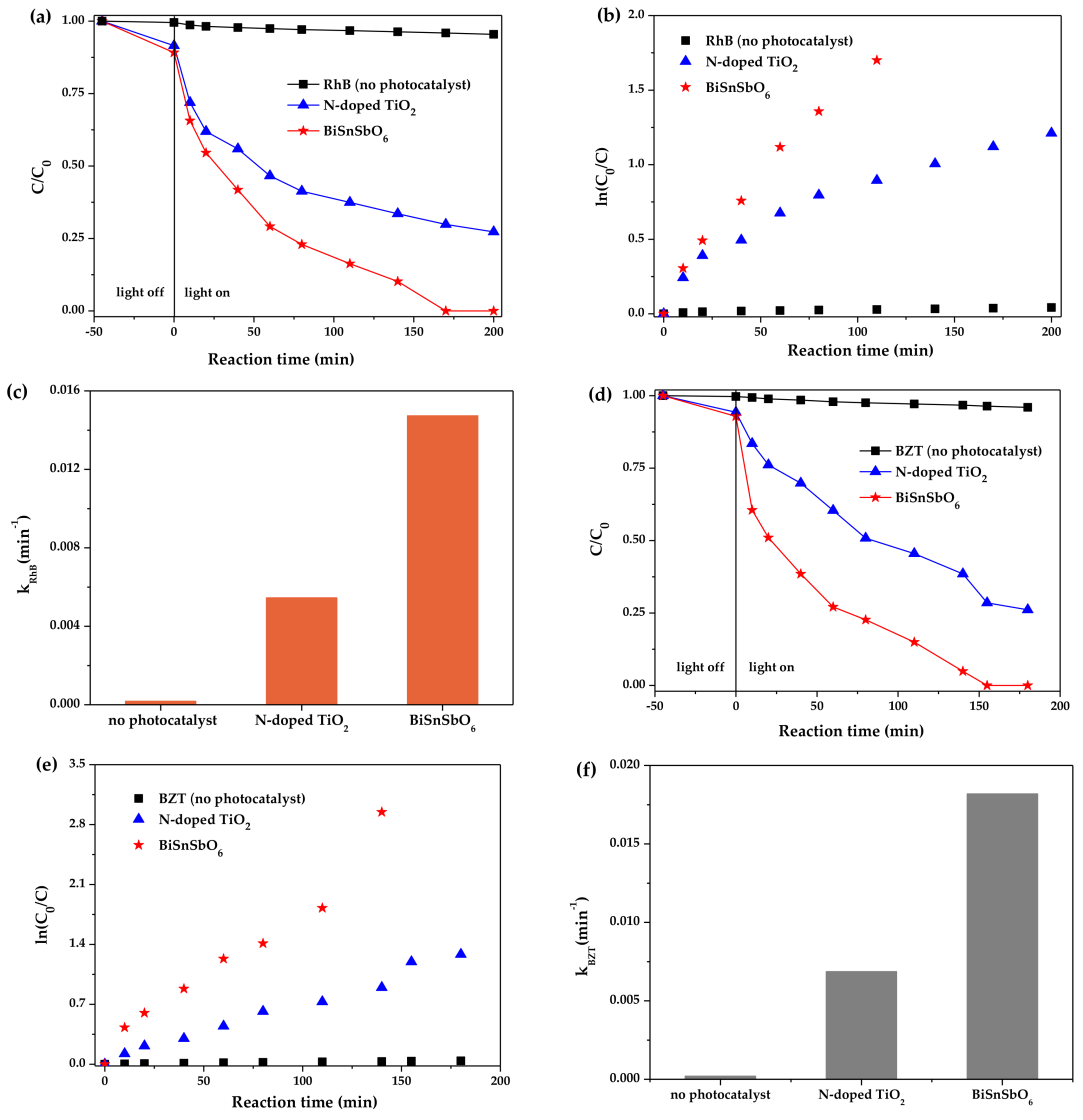
(**a**) Degradation curves of RhB over different samples; (**b**) Linear relation plots of ln(C_0_/C) vs. reaction time; (**c**) The photodegradation rate constants (*k*) calculated from (**b**); (**d**) Degradation curves of BZT over different samples; (**e**) Linear relation plots of ln(C_0_/C) vs. reaction time; (**f**) The photodegradation rate constants (*k*) calculated from (**e**).

The photodegradation curves of RhB and BZT over different photocatalysts are shown in Figure 6. From Figure 6, the initial rate and the initial photon efficiency of the RhB and BZT degradation under visible light irradiation with BiSnSbO_6_ as catalyst were measured, respectively. The surveys were in progress under oxygen saturation conditions ([O_2_]_sat_ = 1.02 × 10^−3^ M). After stirring in the darkness for 45 min, the suspension had already achieved adsorption/desorption equilibrium. With the absence of the photocatalysts, the RhB and BZT were scarcely reduced under simulated solar irradiation. Under visible light irradiation for 200 min, the removal rate of RhB was estimated to be 72.72% with N-doped TiO_2_ as catalyst, while BiSnSbO_6_ could attain a photodegradation efficiency of 100%. Here, the initial photodegradation rate of RhB was 2.398 × 10^−9^ moL·L^−1^·s^−1^ and the initial photon efficiency was found to be 0.0501% (*λ* = 420 nm) for BiSnSbO_6_. In contrast, the photocatalytic degradation ratio with BiSnSbO_6_ as catalyst was better than that with N-doped TiO_2_ as catalyst. Under the same reaction conditions for 200 min, the initial photodegradation rate of RhB was 1.417 × 10^−9^ moL·L^−1^·s^−1^ and the initial photon efficiency was 0.0298% for N-doped TiO_2_. Meanwhile, as shown in Figure 6d, under visible light irradiation for 200 min over N-doped TiO_2_, the initial photodegradation rate of BZT and the initial photon efficiency were respectively found to be 1.908 × 10^−9^ moL·L^−1^·s^−1^ and 0.0401%. However, the initial photodegradation rate of BZT with BiSnSbO_6_ as a photocatalyst was about 2.568 × 10^−9^ moL·L^−1^·s^−1^, which was 1.35 times greater than that with N-doped TiO_2_ as catalyst, and the initial photon efficiency was found to be 0.0539% (*λ* = 420 nm). Therefore, it could be obtained from above results that BiSnSbO_6_ had better photocatalytic activity than N-doped TiO_2_.

The kinetic results of RhB and BZT degradation under visible light irradiation are inferred and are shown in Figure 6b,c,e,f, which show the kinetic results with BiSnSbO_6_, N-doped TiO_2_ and no photocatalyst. Here, C and C_0_ stood for the RhB (or BZT) concentration at time t and t_0_. The relationship between ln(C/C_0_) and the irradiation time was linear correlation. Assuming that the RhB photodegradation process adhered to pseudo first-order decay kinetics, the calculational *k* value for BiSnSbO_6_ was 0.0147 min^−1^, which was 2.7 times greater than the *k* value of 0.0055 min^−1^ for N-doped TiO_2_. As shown in Figure 6f, the first-order rate constant *k* for BiSnSbO_6_ on degrading BZT was found to be 0.0182 min^−1^, while that of N-doped TiO_2_ was 0.0069 min^−1^. It was clear that BiSnSbO_6_ had better activity than N-doped TiO_2_ for both RhB and BZT photodegradation under visible light irradiation.

**Figure 7:**
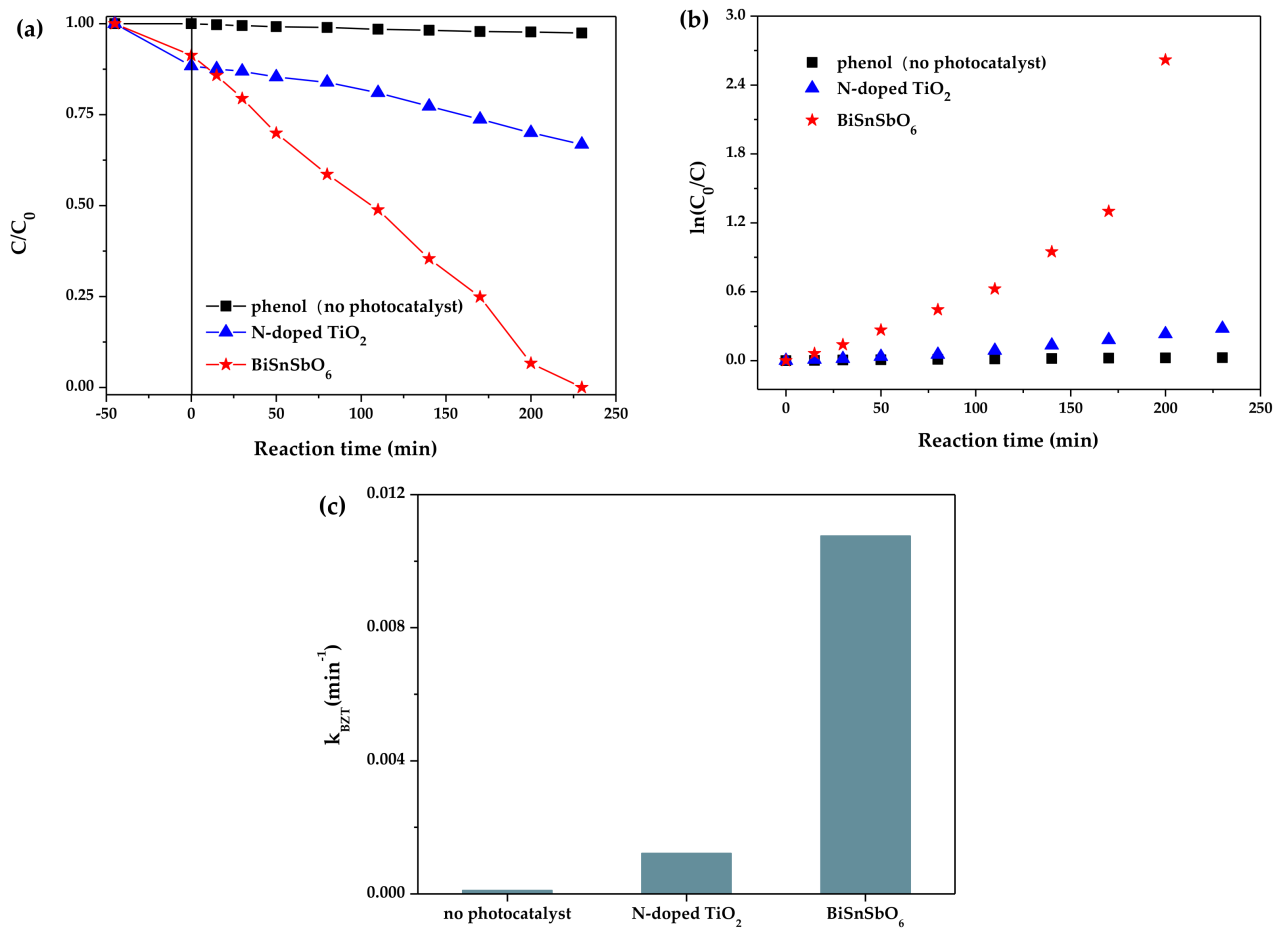
(**a**) Degradation curves of phenol over different samples; (**b**) Linear relation plots of ln(C_0_/C) vs. reaction time; (**c**) The photodegradation rate constants (*k*) calculated from (**b**).

In order to exclude the photosensitization effect under light irradiation, the photocatalytic properties of BiSnSbO_6_ and N-doped TiO_2_ toward the colorless phenol (0.032 mM and 300 mL) are evaluated and the results can be seen in Figure 7. Under visible light irradiation for 230 min, the removal rate of phenol over BiSnSbO_6_ was up to 100%, while that over N-doped TiO_2_ was only 33.16%. As shown in Figure 7b,c, the calculational *k* value of BiSnSbO_6_ for the phenol photodegradation was higher than that of N-doped TiO_2_. Above results demonstrated that the photosensitive effect was not a main factor in the photodegradation process of RhB with BiSnSbO_6_ as a photocatalyst and BiSnSbO_6_ itself had photocatalytic activity [54,55].

In order to compare the photocatalytic activity results of the current system with the data from the literature, the catalytic activity of the photocatalyst was evaluated by using *k*_BZT_/S_BET_ (i.e., degradation rate constant per unit area). Here, *k*_BZT_ was the first-order rate constant *k* for BZT photodegradation, and S_BET_ was the BET surface of the photocatalyst. Table 2 shows the photocatalytic activity results from the literature data and our work for BZT degradation. As shown in Table 2, we could observe that BiSnSbO_6_ showed a slightly higher *k*_BZT_/S_BET_ value compared with BiOBr photocatalyst, indicating that BiSnSbO_6_ is considered a potential photocatalyst to remove BZT contaminant.

**Figure 8:**
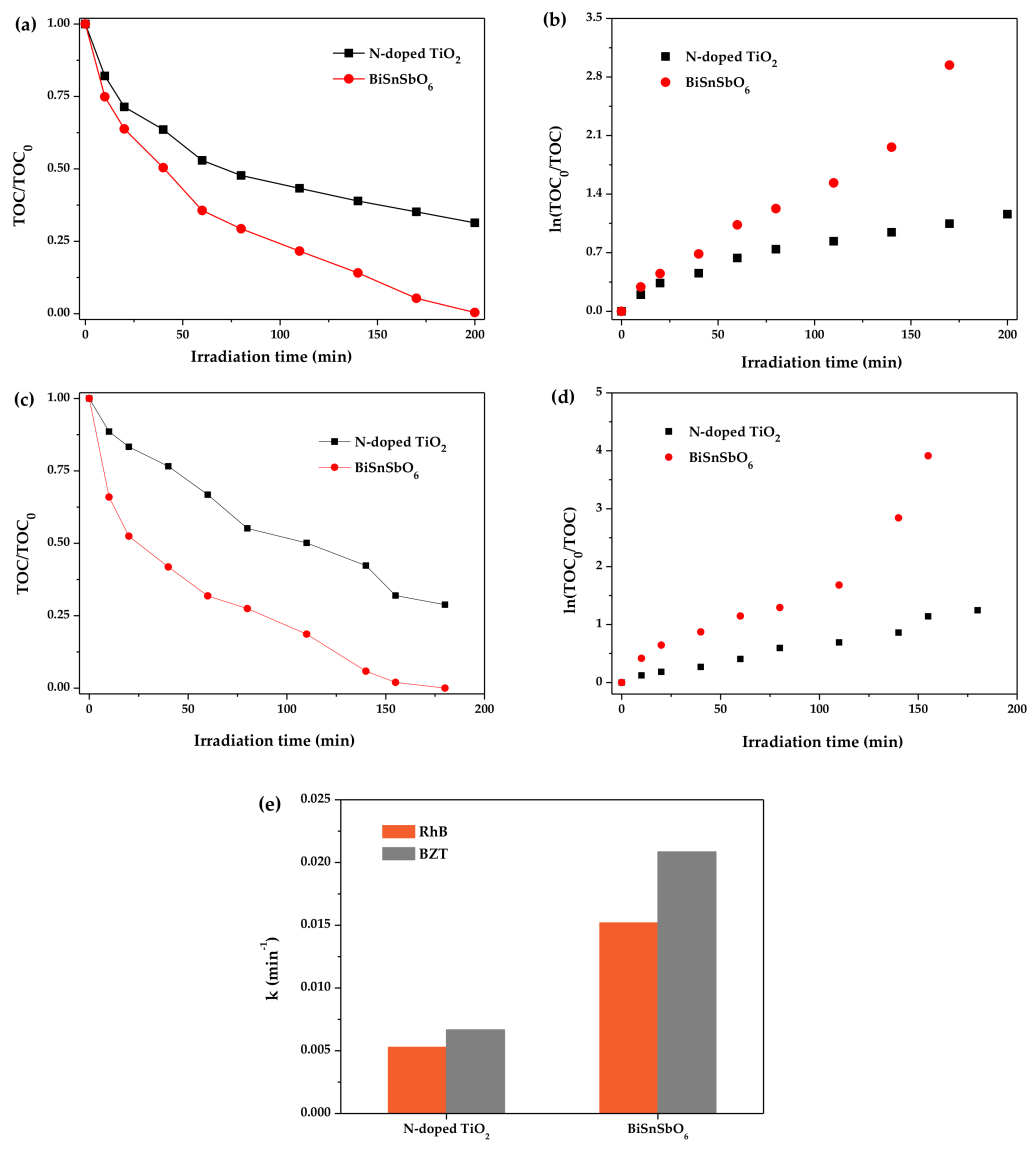
(**a**) Total organic carbon curves of RhB over different samples; (**b**) Linear plots of ln(TOC_0_/TOC) vs. reaction time; (**c**) Total organic carbon curves of BZT over different samples; (**d**) Linear plots of ln(TOC_0_/TOC) vs. reaction time; (**e**) The removal rate constants (*k*) of total organic carbon calculated from (**b**,**d**).

Figure 8a,c describe the removal rate of the total organic carbon in solutions containing BiSnSbO_6_ or N-doped TiO_2_ within 200 min. Figure 8b,d,e show the effect of the irradiation time on the ln(TOC_0_/TOC) and the removal rate constants of TOC. The results showed that the TOC removal rate of RhB reached 99.59% with BiSnSbO_6_ as catalyst, while the TOC removal rate of RhB was only 74.27% with N-doped TiO_2_ as catalyst, after simulated sunlight irradiation for 200 min. Meanwhile, under simulated sunlight irradiation for 180 min, the TOC removal rate of BZT was up to 100% with BiSnSbO_6_ as catalyst, while the TOC removal rate of BZT was only 71.20% with N-doped TiO_2_ as catalyst. Above results showed that the target contaminants (RhB and BZT) were almost completely mineralized when BiSnSbO_6_ was used as a photocatalyst under visible light irradiation. In addition, the intermediate products might be produced during the photodegradation of RhB and BZT. The relationship between the ln(TOC_0_/TOC) and the irradiation time of RhB and BZT photodegradation by using BiSnSbO_6_ or N-doped TiO_2_ could be easily obtained from Figure 8b,d. It was noteworthy that the relationship between ln(TOC_0_/TOC) and irradiation time was linear. In the process of RhB photodegradation, the apparent first-order rate constant *k* for BiSnSbO_6_ was calculated to be 0.0152 min^−1^, which was 2.9 times greater than that of 0.0053 min^−1^ for N-doped TiO_2_. For the BZT photodegradation process, the pseudo first-order rate constant *k* for BiSnSbO_6_ was estimated to be 0.0209 min^−1^, which was 3.1 times greater than that of 0.0067 min^−1^ for N-doped TiO_2_. Above results of photocatalytic degradation indicated that BiSnSbO_6_ could be more effectively excited by visible light than N-doped TiO_2_.

**Figure 9:**
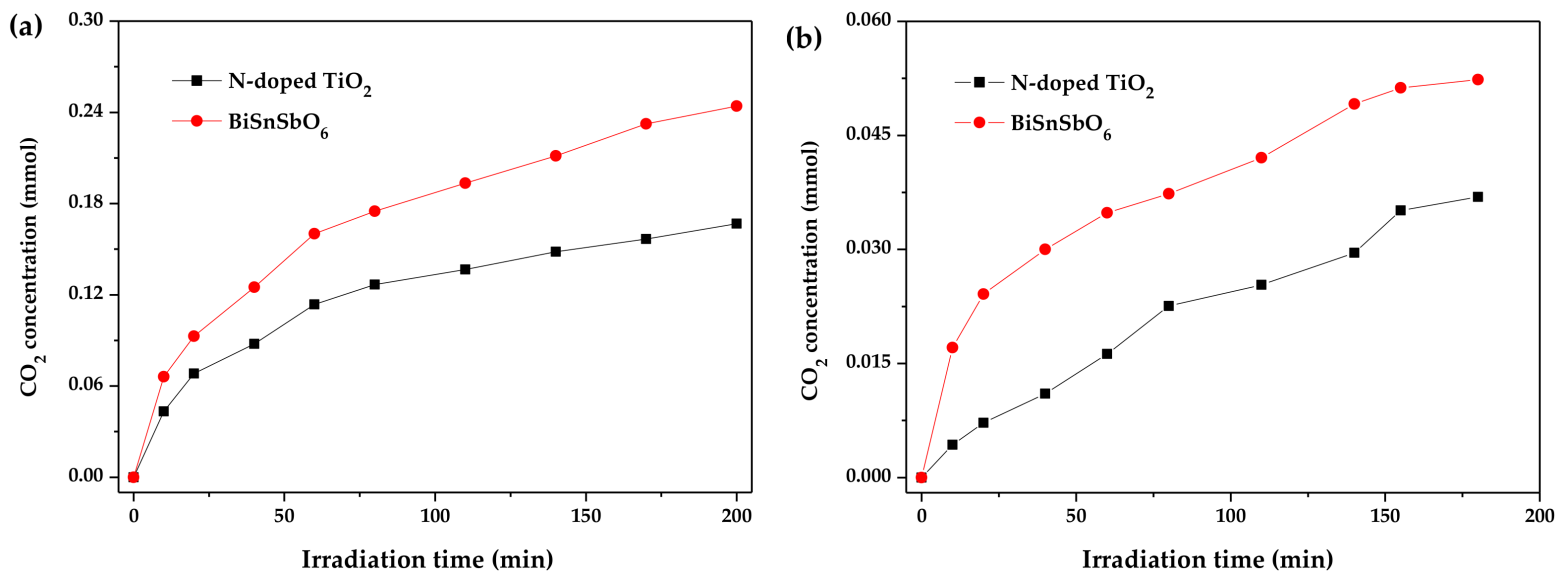
CO_2_ generation during the RhB photodegradation (**a**) and the BZT photodegradation (**b**) over BiSnSbO_6_ or N-doped TiO_2_.

In order to further investigate the mineralization of RhB and BZT during the photocatalytic degradation process over BiSnSbO_6_ or N-doped TiO_2_, the amount of CO_2_ generation was measured. Figure 9 shows CO_2_ generation during the photodegradation of RhB or BZT with BiSnSbO_6_ or N-doped TiO_2_ as catalyst. The results showed that the amount of CO_2_ was gradually increased with the gradual extension of the reaction time. As shown in Figure 9, the rate of CO_2_ production for RhB or BZT photocatalytic degradation with BiSnSbO_6_ as catalyst was faster than that with N-doped TiO_2_ as catalyst. For example, after exposing BiSnSbO_6_, N-doped TiO_2_, RhB and BZT to simulated sunlight for 200 min, the amount of CO_2_ produced from degrading RhB was 0.2441 mmol with BiSnSbO_6_ as catalyst, which was 1.5 times greater than that of 0.1667 mmol with N-doped TiO_2_ as catalyst. The turnover number which was the ratio between total amount of gas produced and photocatalyst in RhB photodegradation, was found to be more than 0.30 for BiSnSbO_6_ and 0.20 for N-doped TiO_2_. Furthermore, after photocatalytic reaction for 180 min under visible light irradiation, the CO_2_ generated during the degradation of BZT was 0.0369 mmol over N-doped TiO_2_, which was 0.7 times greater than that of 0.0523 mmol with BiSnSbO_6_ as catalyst. The turnover number for BZT photodegradation was found to be more than 0.06 with BiSnSbO_6_ as catalyst and 0.04 with N-doped TiO_2_ as catalyst. These turnover numbers fully confirmed the occurrence of catalytic reactions.

**Figure 10:**
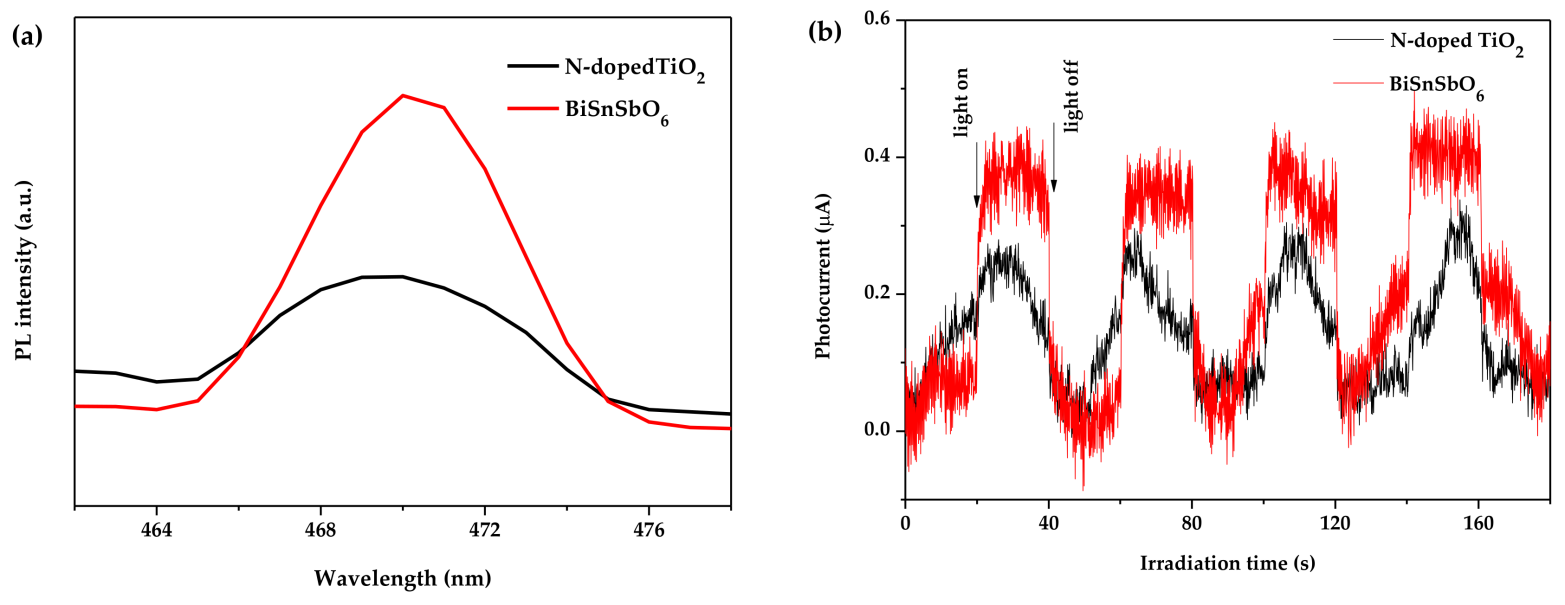
(**a**) The photoluminescence spectra (PL) and (**b**) transient photocurrent response of BiSnSbO_6_ and N-doped TiO_2_.

Figure 10 shows the photoluminescence spectra and transient photocurrent response of BiSnSbO_6_ and N-doped TiO_2_. The photoluminescence spectra patterns of BiSnSbO_6_ and N-doped TiO_2_ in Figure 10a showed a higher photoluminescence spectra intensity of N-doped TiO_2_ than that of BiSnSbO_6_. In general, the higher photoluminescence spectra intensity indicated a higher recombination rate of photogenerated electrons and holes, which had an important effect on the photocatalytic activity [56,57]. However, in this work, BiSnSbO_6_ which owned higher photoluminescence spectra intensity exhibited higher photocatalytic activity than N-doped TiO_2_. Above-mentioned discrepancy might be caused by oxygen vacancies and crystalline defects [58,59,60,61] on the surface of BiSnSbO_6_. During the preparation of BiSnSbO_6_, the volatilization of Bi_2_O_3_ and oxygen vacancies might appear on the surface of the BiSnSbO_6_ samples when the calcination temperature was higher than 820 °C [62]. The photocurrent analysis was also performed to study the interface charge transfer kinetics. As shown in Figure 10b, the transient photocurrent response produced by BiSnSbO_6_ was more clearly observed than that produced by N-doped TiO_2_. Since the high photocurrent intensity indicated highly separable electron–hole pairs and a favourable photocatalytic performance [63], it could be concluded that BiSnSbO_6_ had a more powerful charge separation capacity compared with N-doped TiO_2_. Moreover, the photocurrent intensity rapidly increased when the light was turned on, indicating the apparent photoresponse of BiSnSbO_6_ and N-doped TiO_2_.

**Figure 11:**
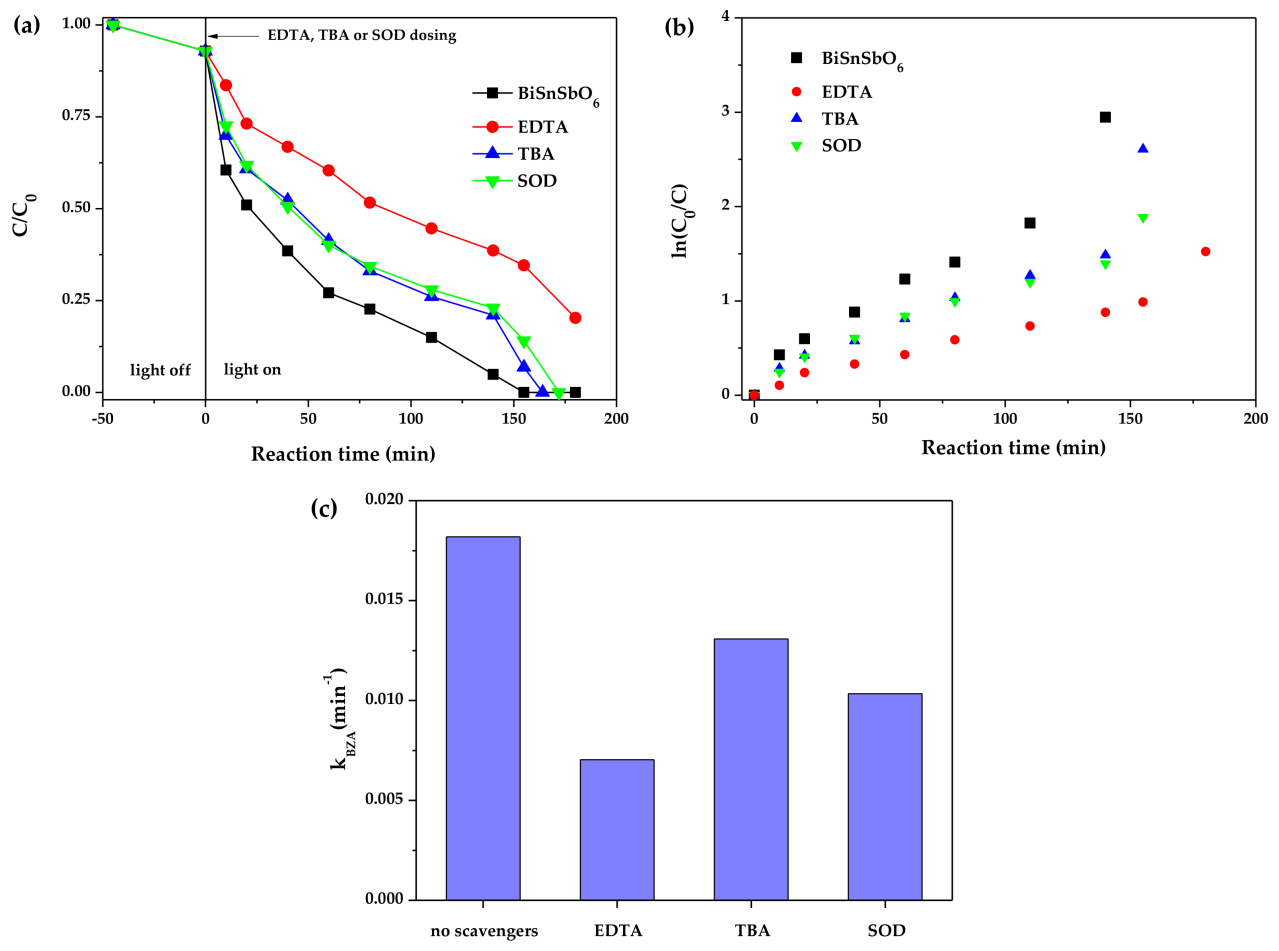
(**a**) Degradation of BZT over BiSnSbO_6_ in the presence of different scavengers (1.5 mM, 2.5 vol% of reaction solution); (**b**) Linear relation plots of ln(C_0_/C) vs. reaction time; (**c**) The photodegradation rate constants (*k*) calculated from (**b**).

Figure 11a shows the degradation of BZT with BiSnSbO_6_ as catalyst in the presence of different scavengers. Figure 11b shows the linear relation curves between ln(C_0_/C) and reaction time. Figure 11c shows the photodegradation rate constants by using different scavengers. As shown in Figure 11, ethylene diamine tetraacetic acid (EDTA), tert-butanol (TBA) and superoxide dismutase (SOD) were used as scavengers to remove h^+^, •OH and •O_2_^−^ [64,65,66] respectively and the main active substances could be further obtained and verified during the photodegradation of BZT with BiSnSbO_6_ as catalyst. For the TBA and SOD photocatalytic systems, the photodegradation efficiency of BZT over BiSnSbO_6_ remained almost the same. Comparing the apparent first-order rate constant *k* calculated from the TBA and SOD photocatalysis system, the *k*_SOD_ of 0.0103 min^−1^ was smaller than *k*_TBA_ of 0.0131 min^−1^, which confirmed that •O_2_^−^ was another main active species. Above active species could be ranked to increase photodegradation rate: •OH < •O_2_^−^ < h^+^.

**Figure 12:**
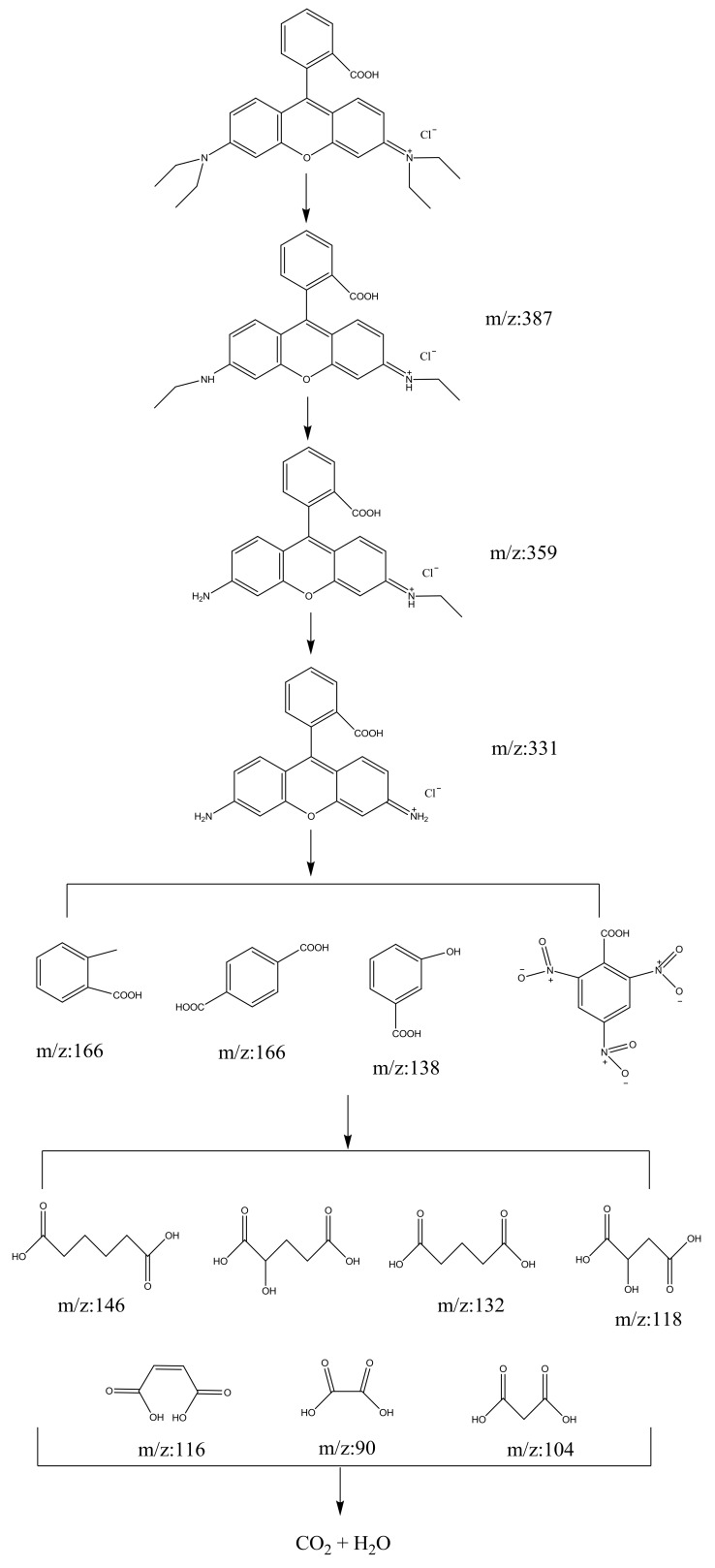
Conceivable photodegradation pathway of RhB under simulated sunlight irradiation with BiSnSbO_6_ as catalyst.

**Figure 13:**
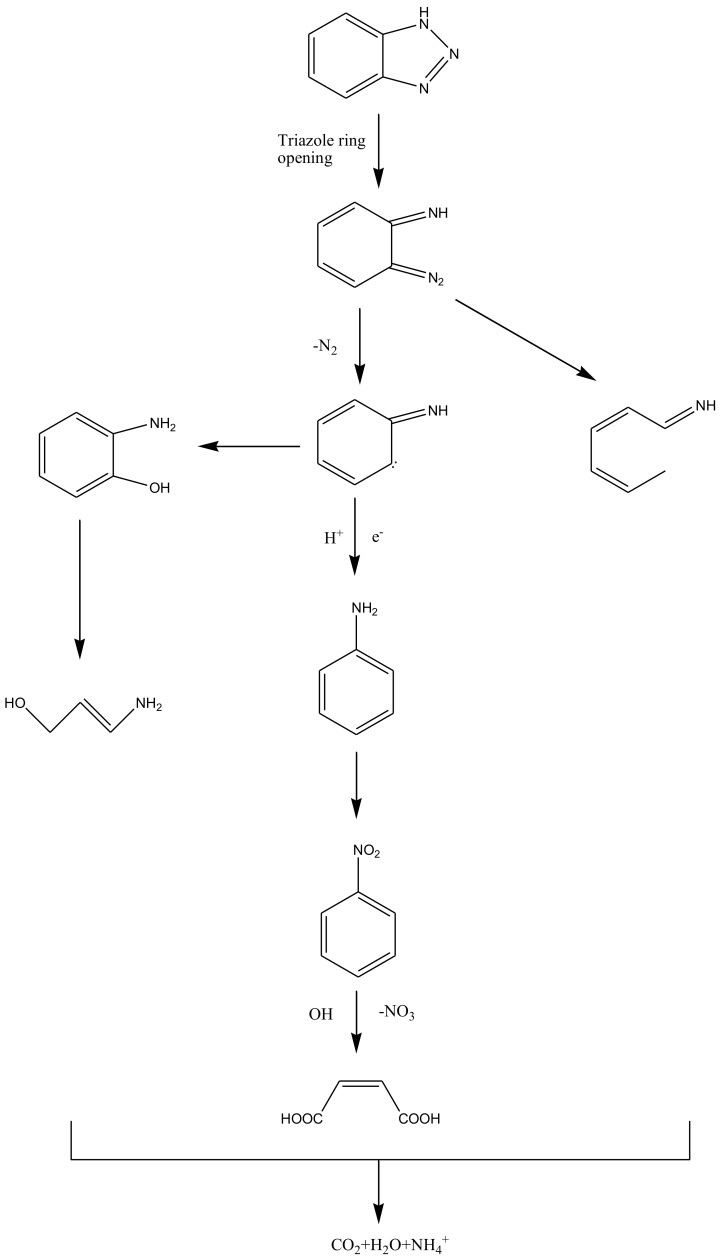
Conceivable photodegradation pathway of BZT under simulated sunlight irradiation with BiSnSbO_6_ as catalyst.

The intermediates of RhB photodegradation in this work were measured as *N*-ethyl-*N*’-ethylrhodamine (*m*/*z*: 387), *N*-ethylrhodamine (*m*/*z*: 359), rhodamine (*m*/*z*: 331), benzoic acid (*m*/*z*: 122), 3-nitrobenzoic acid, 1,2-benzenedicarboxylic acid, terephthalic acid (*m*/*z*: 166), adipic acid (*m*/*z*: 146), 3-hydroxybenzoic acid (*m*/*z*: 138), 2-hydroxypentanedioic acid, pentanedioic acid (*m*/*z*: 132), maleic acid (*m*/*z*: 116), succinic acid (*m*/*z*: 118), malonic acid (*m*/*z*: 104) and oxalic acid (*m*/*z*: 90). Based on these photocatalytic intermediates, a probable photodegradation pathway of RhB is shown in Figure 12. This pathway was similar to, but not the same as the pathway presented by Horikoshi et al. [67] or our previous work [48]. In the research which came from Li et al. [68], the photocatalytic degradation of RhB included two major complex processes: the *N*-demethylation process and the destruction of the conjugate structure. In this work, the photodegradation process of RhB was mainly controlled by chromophore decomposition, ring opening and mineralization. In addition, according to the detected photodegradative intermediates of BZT, a possible photodegradation pathway of BZT is also deduced, as shown in Figure 13. When BiSnSbO_6_ was irradiated by photons whose energy was higher than the band gap energy of BiSnSbO_6_, photogenerated electron–hole pairs were formed on the surface of BiSnSbO_6_ [11]. The oxidation of BZT might be triggered by the combined oxidation of h^+^ and •O_2_^−^, and the opening process of triazole ring was similar to the results reported by Ding et al. [16] and Xu et al. [11]. In summary, RhB and BZT were first converted into small-molecule organic compounds which were eventually combined with other organic active groups to be turned into CO_2_ and water.

As shown in Figure 6a, a slight decrease in RhB degradation was observed under visible light irradiation in the absence of photocatalysts. After reacting for 200 min under visible light irradiation, the initial photodegradation rate of RhB in the absence of photocatalysts was 9.467 × 10^−11^ moL·L^−1^·s^−1^ and the photon efficiency was found to be 0.0020% (*λ* = 420 nm), which was 0.07 times greater than N-doped TiO_2_, and 0.04 times greater than BiSnSbO_6_. The main reason for the photodegradation of RhB without photocatalysts might be due to the direct photosensitization of dye, which was similar to the report from Liu et al. [69]. In addition, under visible light irradiation for 180 min, the initial photodegradation rate of BZT in the absence of photocatalysts was 1.058 × 10^−10^ moL·L^−1^·s^−1^ and the photon efficiency was found to be 0.0022%.

**Scheme 1:**
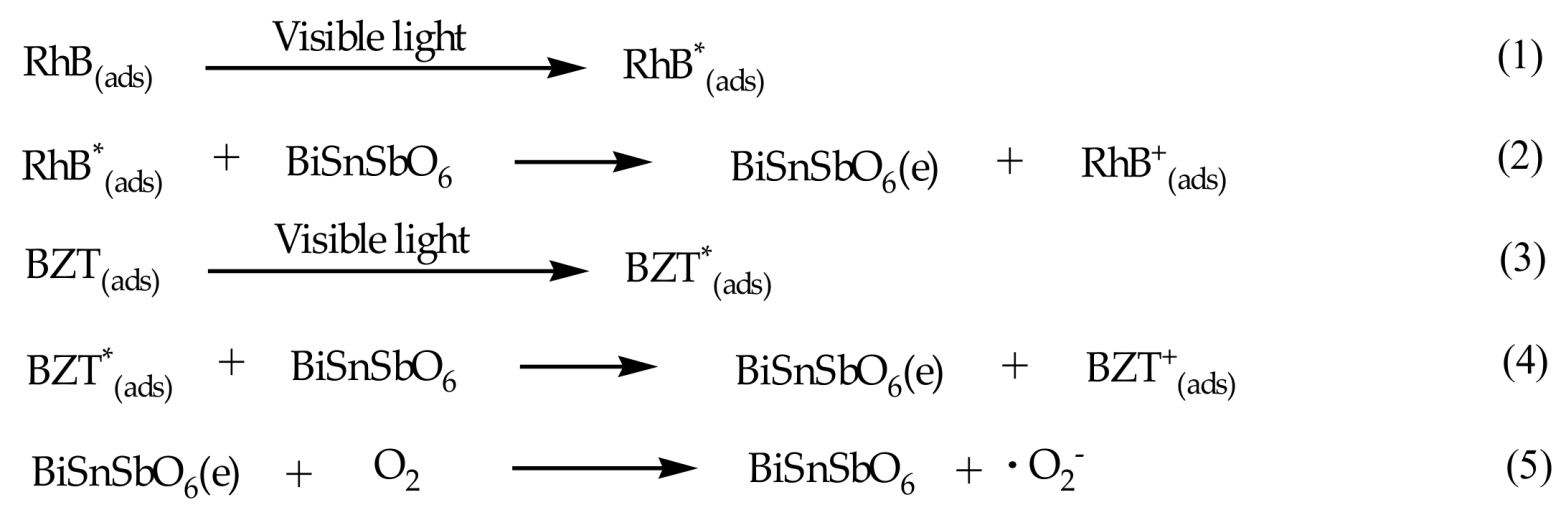
The photosensitization effect on RhB and BZT.

As shown in Figure 4, the apparent photon efficiency at wavelengths which ranged from 440 nm to 800 nm corresponding to sub-E_g_ energy of BiSnSbO_6_ could be easily observed. The photon efficiency without photon assimilation occurred especially between the spectrum in the low energy region and the absorption spectrum of RhB or BZT. Scheme 1 shows the photosensitization effect on RhB and BZT. The result clearly demonstrated that the photocatalytic degradation of RhB or BZT with BiSnSbO_6_ as catalyst at an absorption wavelength which was greater than 440 nm should be attributed to the photosensitization of RhB or BZT itself.

Based on above mechanism, RhB or BZT which was absorbed on the surface of BiSnSbO_6_ was driven by visible light irradiation. Electrons skipped from the excited state of the RhB surface or BZT surface to the conduction band of BiSnSbO_6_. The molecular oxygen and electrons combined to produce superoxide anion. Scheme 1 could explain the results which were gained under visible light irradiation with BiSnSbO_6_ as catalyst, where BiSnSbO_6_ might reduce a recombination of the photogenerated electrons and photogenerated holes via scavenging of the electrons [70].

**Scheme 2:**
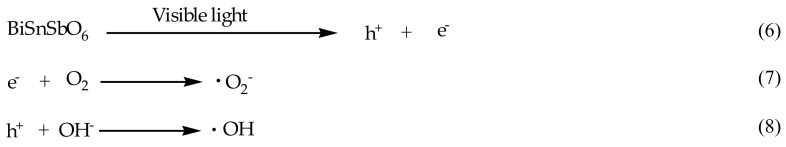
The generation scheme of oxidative radicals with BiSnSbO_6_ as catalyst.

The situation was not the same at absorption wavelength which was below 440 nm. Once the wavelength was below 440 nm, the photon efficiency would correlate intimately with the absorption spectrum of BiSnSbO_6_. In such a case, it was obvious that the band gap excitation of BiSnSbO_6_ was the main mechanism for RhB photodegradation or BZT photodegradation. Although detailed examination of the effects of oxygen and water during the photodegradation of RhB or BZT was not carried out, it was reasonable to conclude that this mechanism was similar to the mechanism which was measured under supra-band gap irradiation. Scheme 2 shows the generation of oxidative radicals with BiSnSbO_6_ as catalyst.

**Figure 14:**
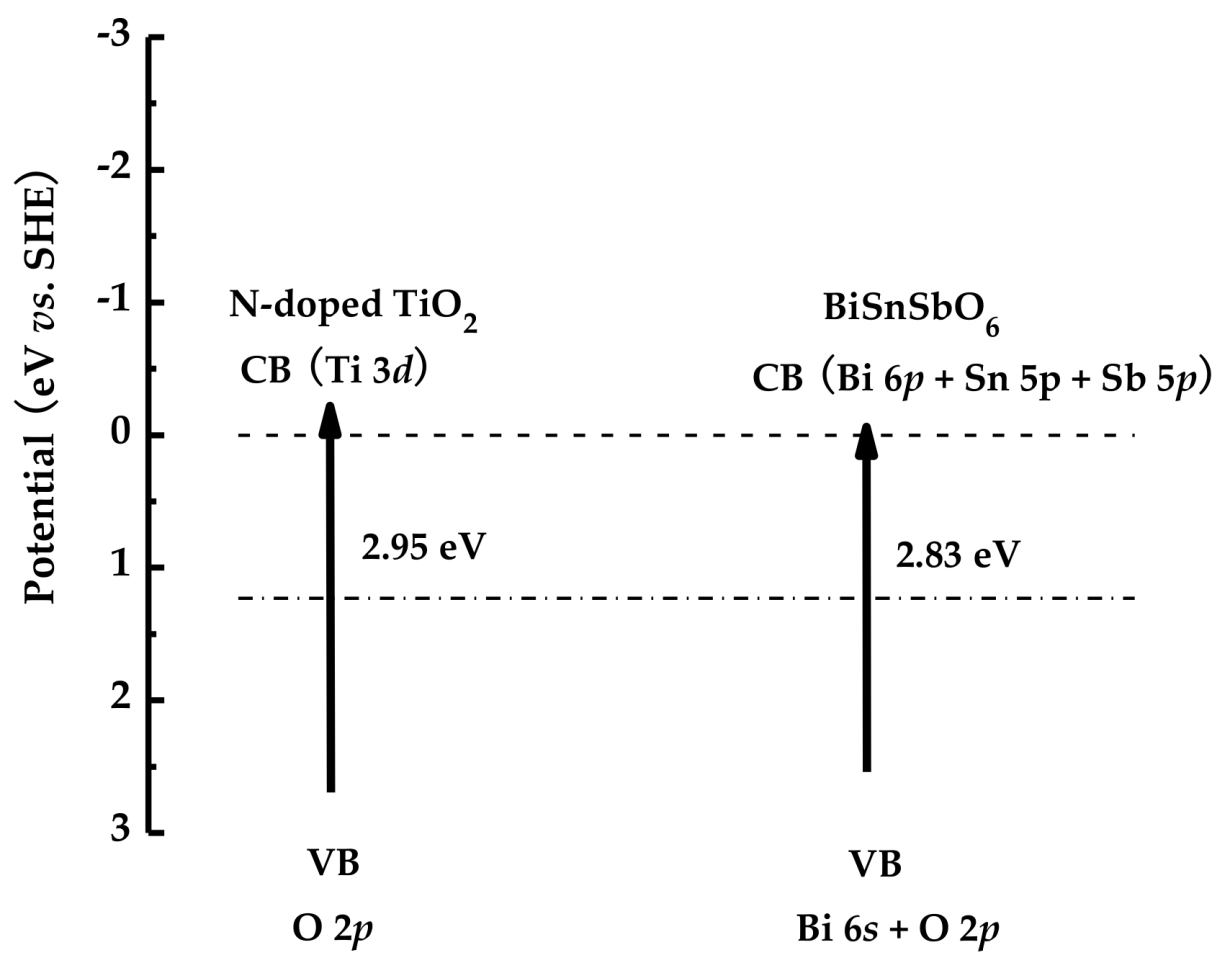
Proposed band structures of BiSnSbO_6_ and N-doped TiO_2_.

Figure 14 shows the proposed band structure of BiSnSbO_6_. The electronic structures of InMO_4_ (M = V, Nb or Ta) and BiVO_4_ were calculated by using the first principles in the work of Oshikiri et al. [71]. This report showed that the conduction band of InMO_4_ (M = V, Nb or Ta) consisted mainly of V 3d orbital component, Nb 4d orbital component and Ta 5d orbital component. The valence band of BiVO_4_ was composed of a partial Bi 6s orbital constituent and a prevailing O 2p orbital constituent. Similarly, the band structure of BiSnSbO_6_ should be similar to that of InMO_4_ (M = V, Nb or Ta) and BiVO_4_. Therefore, the conduction band of BiSnSbO_6_ was constitutive of Bi 6p orbital component, Sn 5p orbital component and Sb 5p orbital component. The valence band of BiSnSbO_6_ consisted of a partial Bi 6s orbital constituent and a prevailing O 2p orbital constituent. Electron–hole pairs were generated in the photocatalyst after BiSnSbO_6_ absorbed photons, indicating that plenitudinous energy which was higher than the band gap energy of BiSnSbO_6_ was essential for the photodecomposition of RhB and BZT.

**Figure 15:**
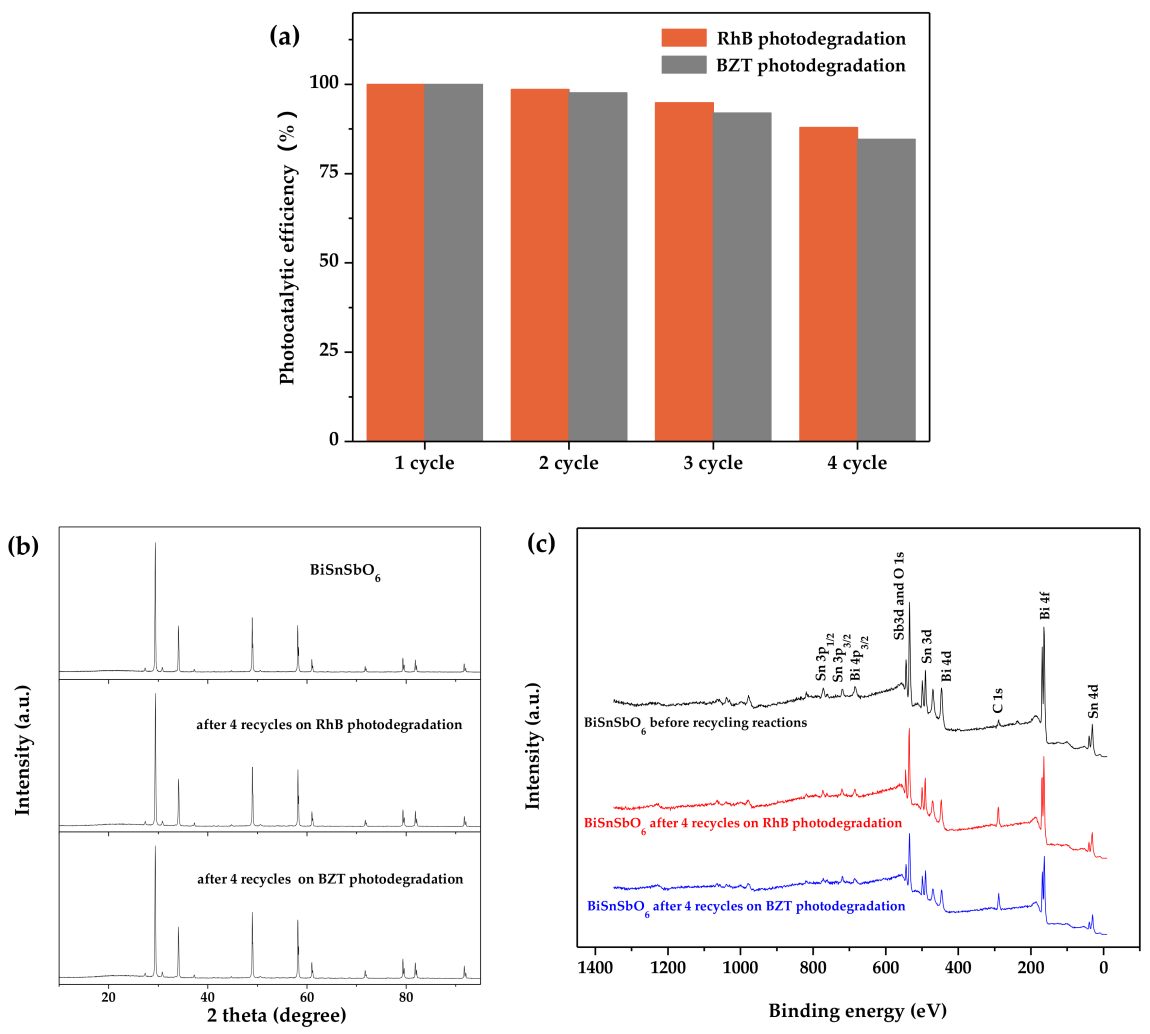
(**a**) The photocatalytic efficiency of RhB degradation or BZT degradation at different recycling time with BiSnSbO_6_ as catalyst.; (**b**) XRD spectra of BiSnSbO_6_ after four recycles of RhB photodegradation or BZT photodegradation; (**c**) XPS spectra of BiSnSbO_6_ after four recycles of RhB photodegradation or BZT photodegradation.

The stability and recyclability of the photocatalysts are important factors for the practical application of photocatalytic materials. Recycling experiments, XRD analysis and XPS analysis were performed to evaluate the stability and recyclability of BiSnSbO_6_. Figure 15a shows the photocatalytic efficiency of RhB degradation or BZT degradation at different recycling time with BiSnSbO_6_ as catalyst. It could be seen from Figure 15a that the photocatalytic efficiencies of RhB over BiSnSbO_6_ under the condition of four cycles were respectively 100% (first cycle), 98.53% (second cycle), 94.84% (third cycle) and 87.88% (fourth cycle). The photocatalytic degradation rates of BZT over BiSnSbO_6_ under the condition of four cycles were respectively 100% (first cycle), 97.63% (second cycle), 91.94% (third cycle) and 84.63% (fourth cycle). Figure 15b shows the XRD spectra of BiSnSbO_6_ after four recycles of RhB photodegradation or BZT photodegradation. The XRD spectra patterns of BiSnSbO_6_ in Figure 15b showed the unchanged phase and structure of BiSnSbO_6_. Figure 15c shows the XPS spectra of BiSnSbO_6_ after four recycles of RhB photodegradation or BZT photodegradation. It could be seen from Figure 15c that the XPS spectra of BiSnSbO_6_ which was gained before and after four recycling reactions revealed that BiSnSbO_6_ had excellent stability and high performance under the condition of photodegradation.

Above results showed that a BiSnSbO_6_/(visible light) photocatalytic system was an effective method to deal with diluted colored wastewater or surface water polluted by BZT-UVs. The BiSnSbO_6_/(visible light) photocatalytic system in this work could be widely used in decolorization, detoxification and purification in the textile, printing and dyeing industries because above system did not demand heating, high pression of oxygen and chemical reagents.

## 4. Conclusions

BiSnSbO_6_ with high photocatalytic activity was first prepared by a high-temperature, solid-state sintering method. The photophysical properties and the photocatalytic properties were investigated via SEM, TEM, XRD, UV-vis and XPS. The results showed that BiSnSbO_6_, with a pyrochlore structure and a cubic crystal system by the space group Fd3m, was well crystallized and was a single phase. The lattice parameter and the band gap of BiSnSbO_6_ were 10.234594 Å and 2.83 eV, respectively. Furthermore, BiSnSbO_6_ showed significant optical absorption in the visible light region (*λ* > 400 nm). BiSnSbO_6_ had a higher photocatalytic activity for degrading BZT and RhB than N-doped TiO_2_ under visible light irradiation. The complete removal of organic carbon could be achieved by measuring the total organic carbon in the presence of BiSnSbO_6_. According to scavenger experiments, during the photocatalytic process the main active species were arranged in order of increasing photodegradation rate: •OH < •O_2_^−^ < h^+^. Thence, it could be concluded that a BiSnSbO_6_/(visible light) photocatalytic system might be a potent method for treating colored wastewater or surface water polluted by BZT-UVs. Finally, the possible photodegradation pathways for RhB and BZT were speculated.

## Figures and Tables

**Table 1:** The binding energies of the element characteristic peaks in the BiSnSbO_6_ photocatalyst.

BiSnSbO_6_	Bi 4f_7/2_	Sn 3d_5/2_	Sb 3d_5/2_	O 1s	C 1s
Binding energy (eV)	163.88	490.98	533.78	534.78	289.08
corrected binding energy of C (eV)	159.8	486.9	529.7	530.7	285.0

**Table 2:** The photocatalytic activity results from the literature data on BZT degradation compared with this work.

Catalyst	Degradation Method	Light Source	Target Pollutant	Removal Rate	S_BET_ (m^2^/g)	*K*_BZT_/S_BET_ (g·min^−1^·m^−2^)		Ref
liquid phase deposited TiO_2_ film	photoelectro- chemistry	15 W UV lamp	0.2 mM benzotriazole	89.8% (180 min)	-		-	[17]
TiO_2_ loaded on Fe^II^Fe_2_^III^O_4_@C	photocatalysis	UV-C lamp, 6 W PHILIPS	60 mg/L (0.5 M) benzotriazole	55.3% (60 min)	269.5		-	[18]
BiOBr	photocatalysis	800 W xenon lamp	1 mg/L (8.4 mM) benzotriazole	89.7% (180 min)	5.86		2.18 × 10^−3^	[12]
BiSnSbO_6_	photocatalysis	500 W xenon lamp	0.032 mM benzotriazole	100% (180 min)	4.78		3.08 × 10^−3^	[our own work]
